# Recent advances in the biosynthesis of polysaccharide-based antimicrobial glycoconjugate vaccines

**DOI:** 10.3389/fmicb.2024.1457908

**Published:** 2025-01-29

**Authors:** Yuhui Wang, Haodi Liu, Baoying Wang, Gülzire Gheyret, Jingliang Qin, Hanlin Wang, Yuhan Di, Yanling Wang, Juan Wang, Haining Tan

**Affiliations:** ^1^National Glycoengineering Research Center, Shandong University, Qingdao, China; ^2^NMPA Key Laboratory for Quality Research and Evaluation of Carbohydrate-Based Medicine, Shandong University, Qingdao, China; ^3^Shandong Provincial Technology Innovation Center of Carbohydrate, Shandong University, Qingdao, China; ^4^School of Pharmaceutical Sciences, Shandong University, Qingdao, China; ^5^School of Life Sciences, Shandong University, Qingdao, China; ^6^Key Laboratory of Molecular Microbiology and Technology, Ministry of Education Nankai University, Tianjin, China; ^7^Jinan Maternity and Child Care Hospital, Jinan, China

**Keywords:** glycoconjugate vaccine, biosynthesis, optimization method, glycosyltransferase, glycoengineering

## Abstract

Glycoconjugate vaccines are a vital category of effective and safe commercial vaccines that have significantly reduced the global prevalence of drug-resistant bacterial infections. These vaccines are synthesized by covalently linking bacterial polysaccharide antigens to a carrier protein. Given that they produce a stronger and longer-lasting immune response than pure polysaccharides that activate only B cells, glycoconjugate vaccines have become one of the most promising vaccine types. However, the chemical synthesis of glycoconjugate vaccines is complex, costly, and labor-intensive. Therefore, the efficient preparation of biosynthetic glycoconjugates using microbial cell factories has emerged as a highly desirable manufacturing alternative. This review focuses on advancements in the recombinant microbial biosynthesis of glycoconjugate vaccines and summarizes various strategies to optimize their production. It is based on three key aspects: the selection of oligosaccharyltransferase (OST), the use of different vaccine carrier proteins, and the enhancement of key concentrations in the uridine diphosphate (UDP)-sugar supply. Finally, the review highlights technical challenges and discusses future directions for the recombinant synthesis of glycoconjugate vaccines.

## Graphical abstract

Overview of the glycoconjugate vaccine synthesis technologies. a,b. Chemical approach for synthesizing glycoconjugate vaccines. a. Extraction and purification of the LPS/glycan and protein backbone from the bacterium; b. Chemical linkage of the OPS to the protein backbone; c–g. Biosynthesis of glycoconjugate vaccines. c. Biosynthesis of glycoconjugate vaccines using N-linked glycosylation; d. Biosynthesis of glycoconjugate vaccines using O-linked glycosylation; e. Alternative therapeutic conjugates targeting bacterial pathogens; f. Application of different carrier proteins in glycoconjugate vaccines; g. Strategies to optimize the UDP-sugar supply.
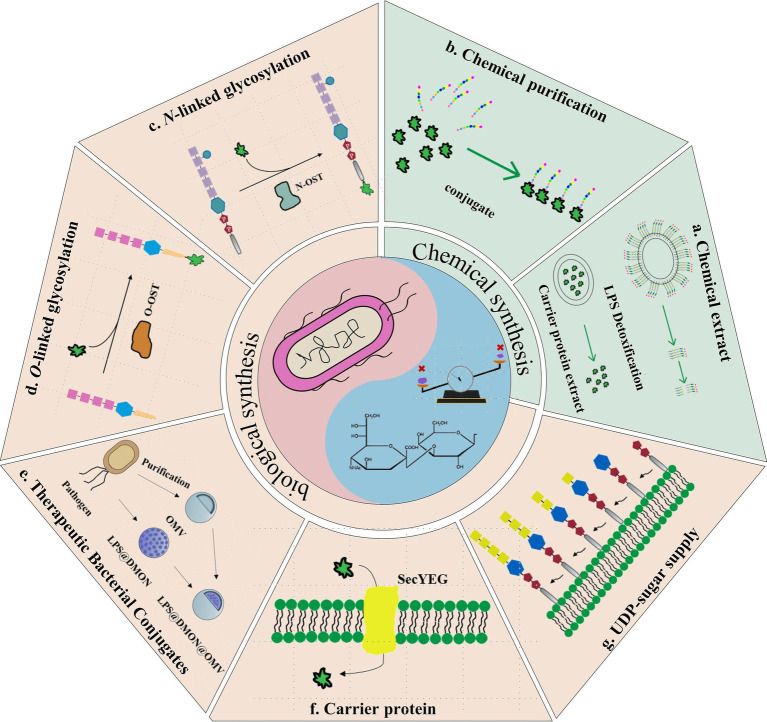

## 1 Introduction

**Figure 1:**
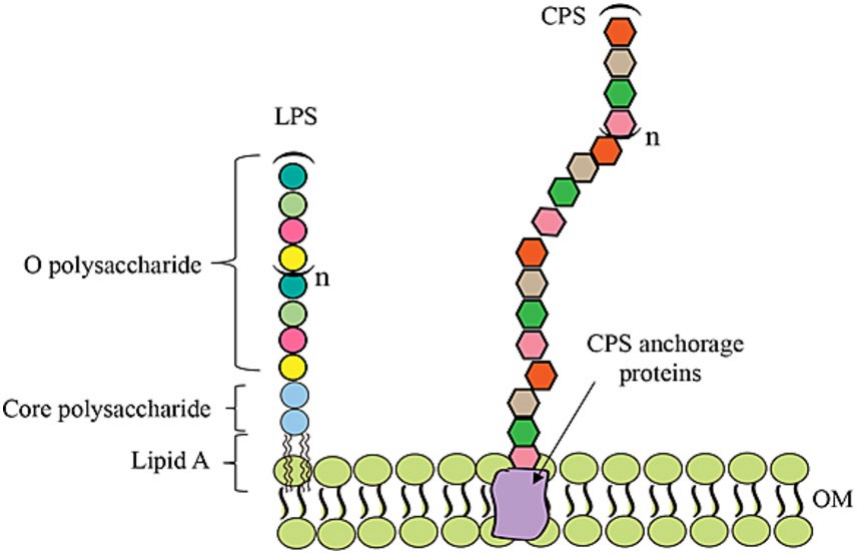
Schematic diagram of the OPS, LPS, and CPS structures in bacteria.

Drug-resistant bacteria are on the rise and pose a major threat, highlighting the urgent need for effective vaccines to prevent infections and save lives (O’Neil, 2014; Pai and Memish, 2016; Micoli et al., 2021; Zhou et al., 2023). Capsular polysaccharides (CPS) or O-antigen polysaccharides (OPSs) are key components of bacterial cells and play significant roles in various biological processes, including inflammation, cellular adhesion, molecular recognition, catalysis, pathogenic infections, and signal transduction events (Figure 1). Given their prominent biological roles, bacterial polysaccharides are promising candidates for use in vaccines. However, pure polysaccharide vaccines can only induce B cells to produce low-affinity IgM, thereby making them ineffective in infants and elderly individuals with immunodeficiencies (Pon and Jennings, 2009; Avci et al., 2011). Glycoconjugate vaccines link a glycan to a protein, resulting in multiple immune system triggers that create long-term immunological memory and increase vaccine stability (Pace, 2013; Micoli et al., 2018b; Xu et al., 2019). In particular, the implementation of fully licensed glycoconjugate vaccines for *Haemophilus influenzae* type b (Hib) (Ladhani, 2012; Perrett et al., 2013), *Neisseria meningitidis* (McCarthy et al., 2018), and some strains of *Streptococcus pneumoniae* (Grijalva et al., 2007) has significantly reduced the occurrence of bacterial meningitis and pneumonia worldwide. In addition, they have contributed to a decrease in the prevalence of antibiotic-resistant infections. Glycoconjugate vaccines provide a significant benefit because they can be effectively and safely administered to a wide range of age groups, including infants and the elderly (Rappuoli, 2018). As a result of the increasing demand for such versatile vaccines, the global glycoconjugate vaccine market was projected to reach approximately US$10 billion by 2020 (Kay et al., 2019).

Global vaccination rates for conjugate vaccines in children are still approximately 30%, with limited access and insufficient immunization coverage contributing to most of the ongoing disease burdens (Wahl et al., 2018). In recent years, the demand for therapeutic and diagnostic glycoconjugates—such as those based on polysaccharides used for *pneumonia* and *meningitis*—has significantly increased. However, progress in their development and distribution has been slow due to the complex and expensive nature of their production. The conventional process for producing conjugate vaccines involves chemically linking carrier proteins to polysaccharide antigens, which are extracted from extensive cultures of pathogenic bacteria. The production of OPS-based glycoconjugates involves several detailed steps (Wang et al., 2023b): (i) extraction of both the LPS/glycan and the protein backbone from the bacterial source; (ii) thorough purification of the protein backbone alongside the LPS; (iii) detoxification of the LPS through the chemical removal of lipid A, isolating the OPS; and (iv) chemical conjugation of the isolated OPS to the protein backbone. However, there are several drawbacks to large-scale fermentative production. The isolation of polysaccharides from the corresponding pathogenic bacterial serovars always involves safety concerns. Each step of the process incurs considerable losses and is time-consuming, which greatly increases the cost of glycoconjugates and limits their application in developing countries. Moreover, each glycoconjugate synthesis presents unique challenges, requiring a specific conjugation method and an individually designed synthetic scheme for each glycoconjugate.

Following the discovery of glycoconjugate synthesis in bacteria and the successful transfer of glycosylation pathways across species, *Escherichia coli* (*E. coli*) has emerged as a practical model for exploring glycosylation, decoding the glycan structures of living cells, and producing therapeutic glycoconjugates (Merritt et al., 2013). The use of recombinant *E. coli* as a host for glycoconjugate production has shown considerable promise, with significant developments (Jaffé et al., 2014). Therefore, the biosynthesis of glycoconjugate vaccines is often of interest to synthetic biologists.

Here, we review the promising field of biosynthetic glycoconjugate vaccines, focusing on optimizing strategies for the production of polysaccharide-based glycoconjugate vaccines.

## 2 Advances in the biosynthesis of polysaccharide-based glycoconjugates

**Figure 2:**
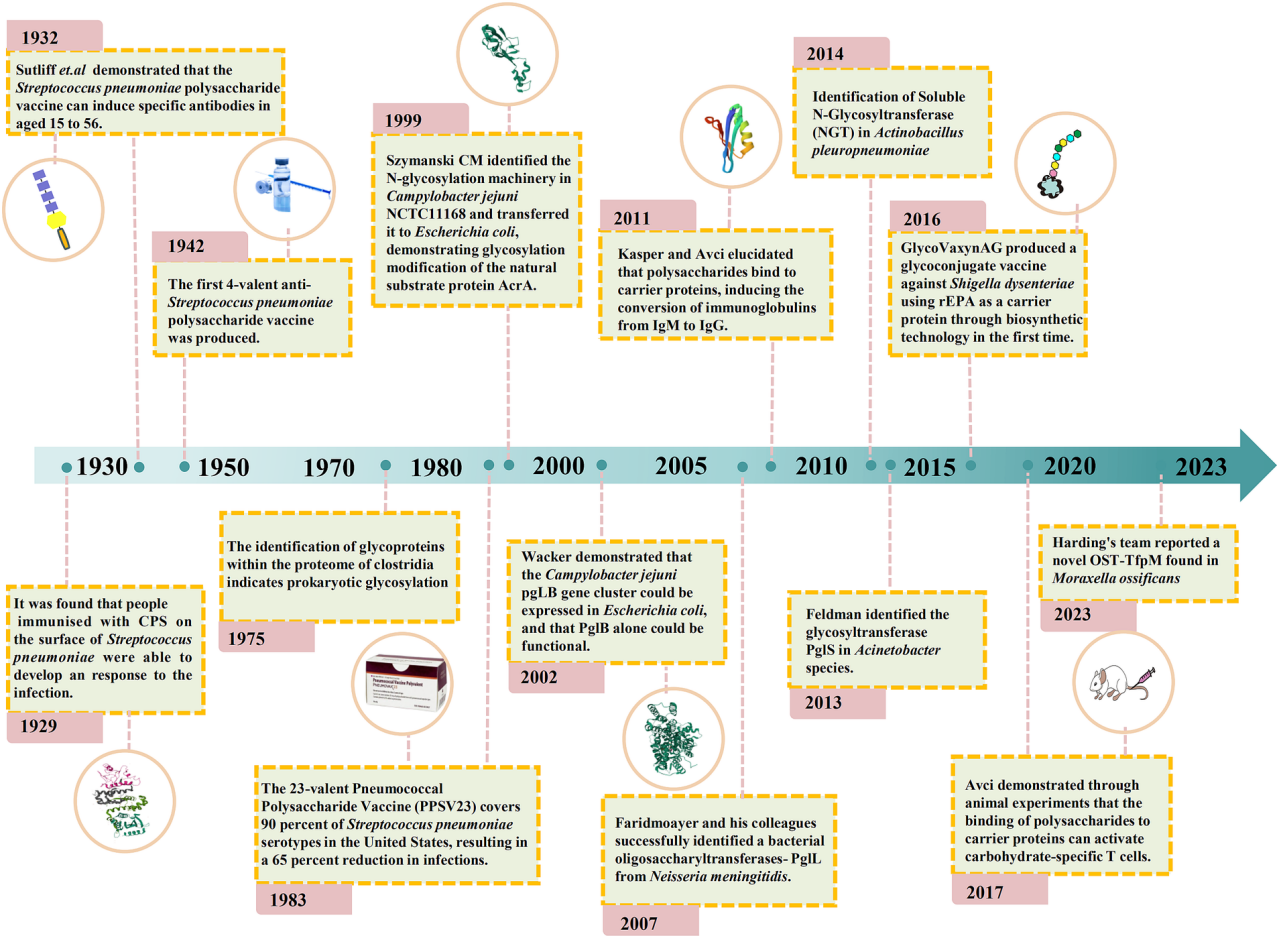
Timeline of key glycoconjugate vaccine technologies and their evolution.

In recent years, there has been a growing interest in developing bacterial species as hosts for glycoengineering applications involving the biosynthesis of structurally diverse polysaccharides, which can be produced as free glycans or as conjugates to carrier proteins (Reid and Szymanski, 2010; Kightlinger et al., 2020). The most obvious advantage of this approach is the much simpler and cheaper culturing conditions required for the maintenance of bacterial cells compared to eukaryotic cell cultures (Schmidt, 2004; Waegeman and Soetaert, 2011; Guarino, 2013). Bacteria carry N- and O-glycosylation systems that are mediated by oligosaccharyltransferase (OST). In OST-dependent glycosylation mechanisms, an oligosaccharide is synthesized on a lipid carrier and subsequently transferred to proteins en bloc by OST. Multiple proteins are glycosylated using this mechanism (Eichwald, 1865; O’Connor and Imperiali, 1996; Wacker et al., 2002). Some unconjugated polysaccharides and glycoconjugates are being biosynthesized as vaccines using microbial cell factories and are currently in the clinical trial phase (Riddle et al., 2016; Huttner et al., 2017). Figure 2 shows the key steps in the history of vaccine technologies and their evolution.

### 2.1 Prokaryotic oligosaccharyltransferase-catalyzed *in vivo* glycosylation of proteins

**Figure 3:**
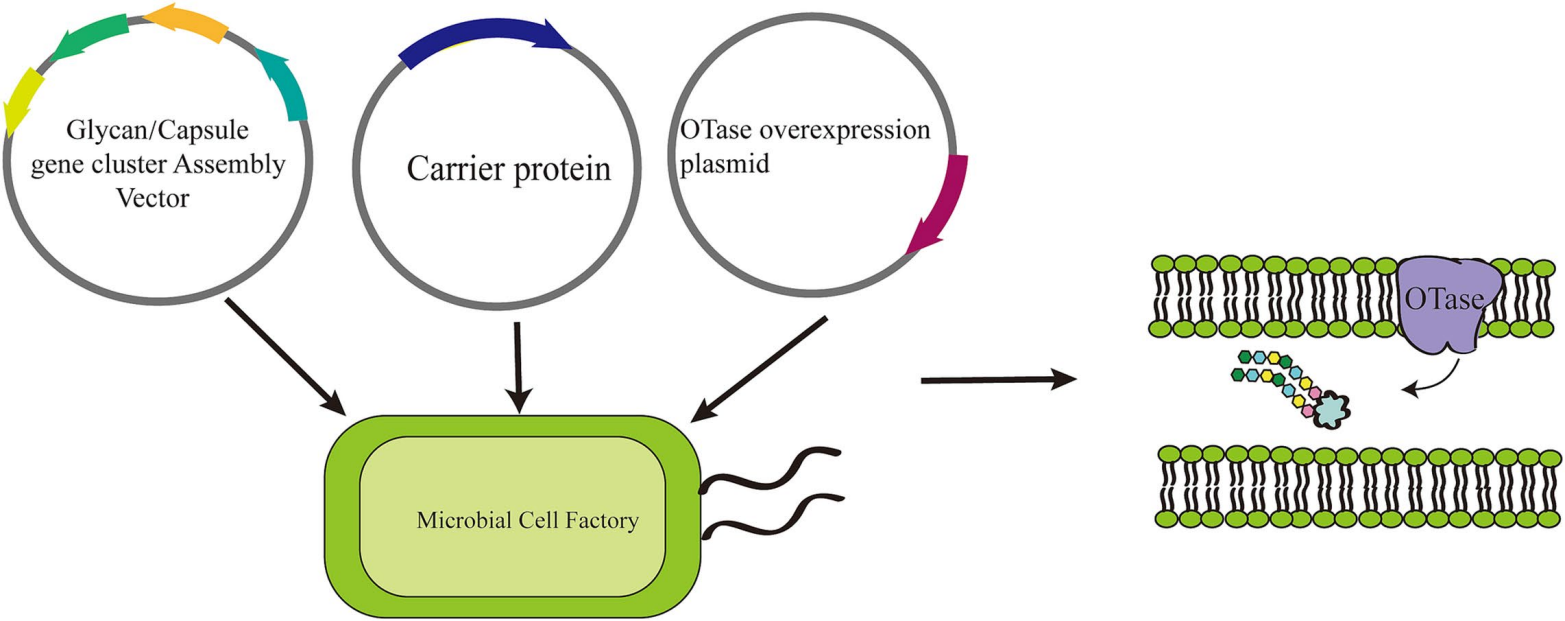
Glycoengineering approach to the production of glycoconjugate vaccines. An *E. coli* cell is engineered with three plasmids to generate glycoconjugate vaccines through a process called Protein Glycan Coupling Technology (PGCT), which unfolds in three distinct stages: polysaccharide expression, carrier protein design and expression, and coupling. Initially, the polysaccharide is synthesized on an undecaprenol pyrophosphate lipid anchor (represented by a blue/black circle) in the cytoplasm. It is then transported to the periplasmic space, where the enzyme PglB recognizes the lipid-linked reducing-end sugar. PglB then transfers the polysaccharide en bloc to an acceptor sequon (D/E-X-N-X-S/T) located on the carrier protein, culminating in the production of the glycoconjugate vaccines. IM refers to the inner membrane, and OM to the outer membrane.

OST selection is a critical consideration in glycosylation, particularly when designing and producing glycoconjugate vaccines and other (Szymanski et al., 1999; Schwarz and Aebi, 2011; Harding and Feldman, 2019; Yakovlieva et al., 2021; Bagdonaite et al., 2022). OST is an enzyme complex responsible for transferring a pre-assembled glycan to specific amino acid residues of nascent proteins (Iwashkiw et al., 2013; Valguarnera et al., 2016). The integration of prokaryotic OST-catalyzed *in vivo* glycosylation into the production pipeline of glycoconjugate vaccines represents a powerful tool for facilitating a critical step in the pathway to generate more effective and accessible vaccines (Figure 3).

#### 2.1.1 Advances in the biosynthesis of polysaccharide-based glycoconjugates using *N*-linked glycosylation

For many years, it was believed that protein *N*-glycosylation occurred exclusively in eukaryotic systems. However, this perception shifted in 1999, when it was discovered that *Campylobacter jejuni* (*C.jejuni*), a Gram-negative bacterium and a pathogen in the human gut mucosa, has a protein *N*-glycosylation apparatus. Subsequent studies found that an OST named CjPglB (PglB from *C. jejuni*) was responsible for glycan transfer to the asparagine side chain in a consensus N-X-S/T sequence of the acceptor protein (Szymanski et al., 1999; Szymanski et al., 2003; Larsen et al., 2004). Notably, CjPglB, a single-subunit protein, was found to be homologous to STT3, the catalytic domain of the multi-subunit eukaryotic OST (Matsumoto et al., 2012).

In 2002, Aebi et al. first reported a bottom-up glycoengineering method using PglB-catalyzed glycosylation to produce glycoconjugate vaccines in *E. coli* (Wacker et al., 2002). Following this concept, several bacterial glycoconjugate vaccines have been biosynthesized using the *N*-linked glycosylation system in *E. coli,* and some of these vaccines have been successfully applied in clinical trials. Table 1 summarizes the glycoconjugate vaccine candidates generated and tested to date. In 2010, Ihssen et al. designed a glycoconjugate vaccine against *Shigella dysenteriae,* which was recently applied in a phase I clinical trial (Ihssen et al., 2010). Urinary tract infections (UTIs) are among the most common bacterial infections in humans. In over 80% of acute, uncomplicated cystitis cases, uropathogenic *E. coli* (UPEC) is the responsible pathogen (Kot, 2019). Indeed, the generation of antibodies targeting the O-antigen has proven to be effective in providing protection against recurrent UTIs caused by *E. coli*. Consequently, a vaccine targeting this antigen is promising due to its demonstrated safety and effectiveness. The promising glycoconjugate vaccine ExPEC4V, which contains O-antigens from UPEC serotypes O1A, O2, O6A, and O25B, was produced and showed positive results in phase II human clinical trials (Huttner et al., 2017). The EXPEC9V vaccine, another conjugate vaccine currently in a phase 3 clinical trial, has also shown promise against UPEC (Saade et al., 2020). In addition, the decavalent conjugate vaccine known as EXPEC10V, which targets a broad spectrum of serotypes (O1, O2, O4, O6, O8, O15, O16, O18, O25B, and O75), demonstrated high effectiveness against invasive extraintestinal *E. coli* in phase 1 clinical trials (Fierro et al., 2023). Hence, recombinant production of glycoconjugates in *E. coli* appears to be a promising alternative to traditional methods used for biomanufacturing conjugate vaccines. Although bacterial-linked OST can transfer a broader array of glycan structures, they still require acetylation at the C2 position of the reducing sugar, which limits the transfer of some glycans (Izquierdo et al., 2009; Ramírez et al., 2017; Napiórkowska et al., 2018).

**Table 1:** Summary of biosynthetic vaccine candidates using various glycosyltransferases in this review, with the potential to prevent bacterial infectious diseases.

Glycosyltransferase	Organism	Carrier protein	Status
PglB	*Shigella dysenteriae* type 1	rEPA	Phase I clinical trials (Ihssen et al., 2010)
	*Shigella flexneri* 2a	rEPA	Phase I clinical trials (Ravenscroft et al., 2019)
	*Francisella tularensis*	rEPA	Laboratory phase (Marshall et al., 2018)
	*Burkholderia pseudomallei*	AcrA	Laboratory phase (Garcia-Quintanilla et al., 2014)
	*Staphylococcus aureus*	rEPA/Hla	Laboratory phase (Wacker et al., 2014)
	*Streptococcus pneumoniae*	AcrA	Laboratory phase (Herbert et al., 2018)
	*Streptococcus pneumoniae*	PiuA	Laboratory phase (Reglinski et al., 2018)
	*Escherichia coli* O1, O2, O6, and O25a	rEPA	Phase I/II clinical trials (Van den Dobbelsteen et al., 2016)
	*Escherichia coli* O1A, O2, O4, O6A, O15, O16, O18A, O25B, and O75	rEPA	Phase III clinical trials (Saade et al., 2020)
	*Escherichia coli* O1A, O2, O4, O6A, O8, O15, O16, O18A, O25B, and O75	rEPA	Phase I/II clinical trials (Fierro et al., 2023)
	*Escherichia coli* O157	MBP	Laboratory phase (Ma et al., 2014)
PglL	*Shigella flexneri*	CTB	Laboratory phase (Pan et al., 2016a)
	*Salmonella para-typhi* A	CTB	Laboratory phase (Sun et al., 2018)
	*Brucella abortus*	CTB	Laboratory phase (Li et al., 2023)
	*Salmonella Typhimurium*	rEPA	Laboratory phase (Shah, 2009)
	*Escherichia coli* O4, O5, O7. and O21	CTB	Laboratory phase (Jiang et al., 2021; Wang et al., 2023a; Wang et al., 2023b)
	*Klebsiella pneumoniae*	CTB	Laboratory phase (Liu et al., 2023b)
PglS	*Streptococcus pneumoniae* 8, 9 V, and 14	rEPA	Laboratory phase (Harding et al., 2019)
	*Klebsiella pneumoniae* K1 and K2	rEPA	Laboratory phase (Feldman et al., 2019)
	*Group B Streptococcus* type Ia, IIb, and III	rEPA	Laboratory phase (Duke et al., 2021)
TfpM	*Escherichia coli* O16	rEPA	Laboratory phase (Knoot et al., 2023)
	*Klebsiella pneumoniae* O2a	rEPA	Laboratory phase (Knoot et al., 2023)
	*Group B Streptococcus type* III	rEPA	Laboratory phase (Knoot et al., 2023)
	*Salmonella enteritidis* LT2	rEPA	Laboratory phase (Lanzilao et al., 2015)

#### 2.1.2 Advances in the biosynthesis of polysaccharide-based glycoconjugates using *O*-linked glycosylation

Over the last decade, in addition to the bacterial *N*-glycosylation mechanism mentioned above, *O*-linked glycosylation that led to the modification of serine or threonine residues has been identified in several bacterial species (Iwashkiw et al., 2013). In contrast to the *N*-linked oligosaccharyltransferase (OST), the *O*-linked OST typically demonstrates more relaxed specificities for glycans while maintaining stricter specificities for acceptor molecules. Four types of bacterial *O*-linked OST such as PilO, PglL, PglS, and TfpM have been utilized in glycobiology. These were first identified in *Pseudomonas aeruginosa*, *Neisseria meningitidis*, *Acinetobacter baylyi*, and *Moraxella osloensis*, respectively (Iwashkiw et al., 2013; Harding and Feldman, 2019; Knoot et al., 2023). In *P. aeruginosa*, PilA has been identified as being modified with a glycan, a modification catalyzed by the glycosyltransferase PilO (Castric, 1995). A similar machinery was found in *N. meningitidis,* where PglL was responsible for the attachment of a carbohydrate moiety to the protein PilE, generating a glycoconjugate (Power et al., 2006). Both PilO and PglL proteins can recognize Und-PP-linked glycans as substrate and tag proteins, demonstrating a promising application of these proteins in the development of glycoconjugate vaccines containing O-linked sugars (Table 2).

**Table 2:** Summary of polysaccharide-based glycoconjugate vaccine candidates using various carrier proteins.

Carrier protein	Organism	Glycan	Coupling method	Status
TT/DT	*Streptococcus pneumoniae*	Capsules - polyvalent(4, 6B, 9 V, 14, 18C, 19F, 23F, 1, 5, and 7F)	Chemical	Marketed (Feldman and Anderson, 2020)
	*Haemophilus influenzae*	PRP	Chemical	Marketed (Lepow et al., 1987)
	*Neisseria meningitidis*	Capsule-serotype A	Chemical	Marketed (Ateudjieu et al., 2020)
	*Neisseria meningitidis*	Capsule-serotype A, C, W, and Y	Chemical	Marketed (Robertson et al., 2023)
	*Neisseria meningitidis*	Capsule-serotype A, C, W, Y, and X	Chemical	Marketed (Robertson et al., 2023)
	*Salmonella Typhimurium*	Capsule-serotype Vi	Chemical	Marketed (Lee et al., 2020)
CRM_197_	*Streptococcus pneumoniae*	Capsules - polyvalent(4, 6B, 9 V, 14, 18C, 19F, and 23F)	Chemical	Marketed (Chibuk et al., 2010)
	*Streptococcus pneumoniae*	Capsules - polyvalent(4, 6B, 9 V, 14, 18C, 19F, 23F, 1, 5, 7F, 3, 6B, and 19A)	Chemical	Marketed (Chibuk et al., 2010)
	*Streptococcus pneumoniae*	Capsules - polyvalent(4, 6B, 9 V, 14, 18C, 19F, 23F, 1, 5, 7F, 3, 6B, 19A, 22F, and 33F)	Chemical	Marketed (Schellenberg et al., 2023)
	*Streptococcus pneumoniae*	Capsules - polyvalent(4, 6B, 9 V, 14, 18C, 19F, 23F, 1, 5, 7F, 3, 6B, 19A, 8, 10A, 11A, 12F, 15B, 22F, and 33F)	Chemical	Marketed (Schellenberg et al., 2023)
	*Neisseria meningitidis*	Capsule-serotype A,C,W, and Y	Chemical	Marketed (Blanchard-Rohner et al., 2013)
	*Haemophilus influenzae*	PRP	Chemical	Marketed (Akeda et al., 2018)
	*Salmonella Typhimurium*	Capsule-serotype Vi	Chemical	Development (van Damme et al., 2011)
rEPA	*Shigella dysenteriae*	O-antigen	Biological	Development (Ihssen et al., 2010)
	*Shigella flexneri*	Capsule- Type 2a	Biological	Development (Ravenscroft et al., 2019)
	*Francisella tularensis*	O-antigen	Biological	Development (Marshall et al., 2018)
	*Escherichia coli*	O-antigen O1, O2, O6, and O25a	Biological	Development (Van den Dobbelsteen et al., 2016)
	*Salmonella Typhimurium*	O-antigen	Biological	Development (Shah, 2009)
	*Streptococcus pneumoniae*	Capsule-serotype 8, 9 V, and 14	Biological	Development (Harding et al., 2019)
	*Klebsiella pneumoniae*	Capsule-serotype K1 and K2	Biological	Development (Feldman et al., 2019)
	*Group B Streptococcus*	Capsule-serotype Ia, IIb, and III	Biological	Development (Duke et al., 2021)
	*Escherichia coli*	O-antigen O16	Biological	Development (Knoot et al., 2023)
	*Klebsiella pneumoniae* O2a	O-antigen O2a	Biological	Development (Knoot et al., 2023)
	*Group B Streptococcus*	Capsule-serotype III	Biological	Development (Knoot et al., 2023)
	*Salmonella enteritidis* LT2	O-antigen LT2	Biological	Development (Lanzilao et al., 2015)
CTB	*Shigella flexneri*	O-antigen	Biological	Development (Pan et al., 2016)
	*Salmonella para-typhi* A	O-antigen	Biological	Development (Sun et al., 2018)
	*Brucella abortus*	O-antigen	Biological	Development (Li et al., 2023)
	*Escherichia coli*	O-antigen O4, O5, O7, and O21	Biological	Development (Jiang et al., 2021; Wang et al., 2023a; Wang et al., 2023b)
	*Klebsiella pneumoniae*	O-antigen O1	Biological	Development (Liu Y. et al., 2023)
MBP	*Escherichia coli*	O-antigen polysaccharide (O157)	Biological	Development (Ma et al., 2014)
AcrA	*Burkholderia pseudomallei*	O-PSII	Biological	Development (Garcia-Quintanilla et al., 2014)
	*Brucella abortus*	O-antigen of *Y. enterocolitica* O9	Biological	Development (Huang et al., 2020)
OMPC	*Haemophilus influenzae*	PRP	Chemical	Marketed (Kniskern et al., 1995)

However, both native PilO and PglL proteins were found to transfer only a single O-antigen subunit rather than longer polysaccharides, which limited their further application. This issue appears to have been recently solved by Pan et al., who elucidated and optimized an *O*-linked “glycosylation tag” as a recognition motif, known as MOOR, for the *O*-glycosyltransferase PglL (Pan et al., 2016). In their research, this recognition motif was successfully fused to both the *N*-terminus and *C*-terminus of different potential carrier proteins, generating glycoconjugate vaccines against *S. typhimurium* and *S. flexneri* 2a pathogen infections, respectively. Inspired by these technological advances, we also added a peptide fragment (^45^SAVTEYYLNHGEWPGNNTSAGVATSSEIK^73^) to the C-terminus of the carrier protein cholera toxin B subunit (CTB) using the PglL-dependent *O*-glycosylation system to generate OPS-based glycoconjugate vaccines against UPEC (Wang et al., 2023a).

Although both the *N*-OST PglB and the *O*-OST PglL exhibit remarkable versatility toward glycan substrates, neither enzyme has been experimentally proven to conjugate glycans containing a glucose residue at the reducing end. However, in approximately 75% of *S. pneumoniae* and many other pathogenic bacteria, CPSs contain glucose as the reducing-end monosaccharide. This indicates that these types of OST are not suitable for the biosynthesis of glycoconjugate vaccines, thereby limiting further application. Nonetheless, the two types of OST, PglS and Tfpm, are now known to transfer glycans with glucose at the reducing end (Harding et al., 2019). PglS was first discovered in *Acinetobacter baylyi* ADP1 and is capable of transferring a diverse array of polysaccharides, including those with glucose as the reducing-end sugar (Harding et al., 2015). Furthermore, Feldman et al. engineered a polyvalent pneumococcal glycoconjugate vaccine using the natural acceptor protein ComP as a vaccine carrier (Feldman et al., 2019; Harding et al., 2019). Several antimicrobial glycoconjugate vaccines using the conventional vaccine carrier *Pseudomonas aeruginosa* exotoxin A protein are already in the clinical trial phase (Porstendörfer et al., 2000; Schulz et al., 2013).

Harding et al. (2019) explored the recognition motif of PglS by fusing a peptide fragment from ComP to the *N*-terminus and *C*-terminus of two vaccine carrier proteins. These proteins included the detoxified variant of diphtheria toxin, CRM197, and recombinant ExoProtein A (rEPA) (Knoot et al., 2021; Knoot et al., 2023). As a result, both proteins were glycosylated. Recombinant *O*-glycoconjugate vaccines were produced with PglS-dependent O-glycosylation against a variety of pathogens, such as *Streptococcus mastitis* and *Klebsiella pneumoniae* (Geno et al., 2015; Pan et al., 2015; Carboni et al., 2017).

In 2023, Harding et al. identified a novel type of *O*-OST, termed TfpM, from *Moraxella* bacteria (Knoot et al., 2023). TfpM proteins are similar in size and sequence to PilO enzymes; however, these proteins can transfer long-chain polysaccharides to acceptor proteins. Furthermore, one of the glycosylation sites on pilin-like proteins is serine (Ser). The ability to tag proteins for TfpM-dependent O-glycosylation expands the potential biotechnological applications of this enzyme family. Utilizing this system, they engineered a variety of glycoconjugate vaccines against bacterial infections.

### 2.2 Alternative therapeutic bacterial conjugates

Although protein glycoconjugation is the most widely studied approach in vaccine research, researchers in the field of bacterial glycobiology are exploring alternative approaches to boost the immunogenicity of carbohydrate epitopes. Nearly all Gram-negative bacteria and some Gram-positive bacteria release outer membrane vesicles (OMVs) during their life cycles (Schwechheimer and Kuehn, 2015). These vesicles are usually nanosized proteoliposomes (ranging in size from 20 to 250 nm) with bilayer membranes that are mainly composed of virulence-associated components (e.g., membrane proteins, CPS, and LPS) (Tan et al., 2018). In light of their immunogenic capacities and high built-in adjuvanticity, OMVs have become promising vaccine candidate antigens (Lei et al., 2019). An OMV-based vaccine derived directly from *N. meningitidis* was developed as a licensed vaccine termed Bexsero® (GlaxoSmithKline), which has proven to be an effective vaccine against serogroup B meningococcal infections (Gorringe and Pajón, 2012). Compared to traditional subunit vaccines, OMV vaccines have numerous advantages: (i) OMVs carry significant amounts of virulence-associated pathogen-associated molecular patterns (PAMPs), which play an essential role in inducing an immune response; (ii) OMVs, as nanoscale particles, enhance the accumulation of antigens in lymph nodes, thereby boosting immunogenicity; and (iii) nanocarriers provide efficient adjuvanticity and stimulate antigen-presenting cell activation to elicit robust immune responses.

A novel bacterial glycoengineering approach to develop OMV-based nanovaccines was reported (Morelli et al., 2021; Long et al., 2022). Inspired by these technological advances, a series of *E. coli*-derived glycosylated OMVs (glycOMVs) were generated (Valguarnera and Feldman, 2017; Xie et al., 2022). These glycOMVs, carrying O-antigens from eight bacterial species, including *F. tularensis* and the CPS of *S. pneumoniae* serotype 14 (CPS14), were shown to elicit significant serum titers of class-switched, glycan-specific IgG antibodies in mice (Price et al., 2016). Notably, mice immunized with glycOMVs decorated with the CPS14 of *S. pneumoniae* elicited the same level of antigen-specific serum titers as mice vaccinated with the commercially licensed glycoconjugate vaccine Prevnar13®. These results indicate that the use of bacterial OMVs decorated with heterologous antigens holds great potential in the design of effective antibacterial vaccines.

In another investigation, a nanoconjugate vaccine was generated using a nano-B5 self-assembly system that carries the O-polysaccharide from *K. pneumoniae* (Pan et al., 2020). This nanovaccine has been shown to effectively boost antigen uptake by antigen-presenting cells and provoke a humoral immune response against *K. pneumoniae*. The designed nano-B5 self-assembly system in this study can effectively integrate various modular components and antigen cargos to efficiently create a potentially vast array of nanovaccine structures using multiple bacterial species.

Furthermore，to explore new areas within the structural domain of glycans and proteins in *E. coli*, Tytgat et al. (2019) engineered a cytoplasmic glycoengineering system to generate a nanoscale glycoconjugate. In their work, the shift from *en bloc* glycosylation to sequential glycosylation was a significant change in methodology. Sequential glycosylation in the cytoplasm allows for a more tailored, stepwise addition of glycan moieties directly to proteins. Moreover, the glycoengineering process occurred in the cytoplasm, marking a groundbreaking approach to protein glycosylation. This innovative approach could potentially enable new functionalities in proteins, enhance the stability and efficacy of therapeutic proteins, and allow for the production of glycoconjugates for diverse future biomedical applications.

### 2.3 Carrier proteins as a vaccine design parameter

Four carrier proteins have been used in licensed bacterial vaccines that promote a T cell-dependent (TD) immune response: tetanus toxoid (TT), diphtheria toxoid (DT), Cross Reactive Material 197 (CRM_197_), and *Haemophilus* protein D (PD) (Giannini et al., 1984; Micoli et al., 2018; David et al., 2019; Ravenscroft et al., 2019; Del et al., 2022). Diphtheria and tetanus toxoids were initially selected as carrier proteins for Hib conjugate vaccines because of their long history of safety and efficacy (Prymula et al., 2006; Forsgren et al., 2008). Immunization of mice with DT/TT/CRM_197_ prior to CRM_197_-conjugated *N. meningitidis* serogroup A and C polysaccharides was found to significantly improve anti-polysaccharide IgG titers (Terra et al., 2012; Moeller, 2022). Additional experiments showed that the activation of carrier protein-specific T helper cells could result in more effective activation of glycan-specific B cells, with carrier-derived fragments presented on their surface (Adamo et al., 2012; Oleksiewicz et al., 2012; Saggy et al., 2012; Zhang et al., 2013).

#### 2.3.1 New protein carriers under investigation

In addition to the carrier proteins already used in licensed commercial glycoconjugate vaccines, many others have been tested in preclinical studies and clinical trials with significant results. The recombinant protein rEPA has been engineered as a carrier to chemically conjugate with *Shigella* O-antigens, *Staphylococcus aureus* CPS5 and CPS8, and *Salmonella* Typhi Vi antigen (Szu et al., 1987; Brakke, 1992; Fattom et al., 1993; Cohen et al., 1997; Kossaczka et al., 1999). These glycoconjugate vaccines have been shown to boost vaccine efficacy. The cholera toxin B subunit (CTB) is a non-toxic pentameric moiety of cholera toxin (CT) and can be safely administered through various routes to humans (Hol et al., 1995; Sanchez and Holmgren, 2008; Baldauf et al., 2015). It has the capacity to induce an antigen-specific serum IgG response, along with toxin-neutralizing immunity. Recently, the CTB has been successfully used as a carrier protein by conjugating antigens to induce immune responses against several pathogens (such as *C. trachomatis*, *H. pylori*, *S. paratyphi A*) (McKenzie and Halsey, 1984; Vempati, 2014; He et al., 2022). Therefore, the CTB is a promising carrier that can be utilized in the development of glycoprotein vaccines.

#### 2.3.2 Proteins with a dual role of a carrier and an antigen

In some cases, prior or simultaneous exposure to a protein can lead to vaccine interference, thereby decreasing glycoconjugate efficacy (Dagan et al., 2010; Borrow et al., 2011). To overcome unwanted vaccine interference, new carrier candidates from different pathogens have been researched at the preclinical level (Micoli et al., 2019; Gebre et al., 2021). Some protein carriers serve a dual role of both a carrier and a protective antigen to elicit or enhance immune responses. Group B *Streptococcus* (GBS) pili proteins GBS80 and GBS67, previously selected as pathogen-derived protein carriers and shown to confer protection, were conjugated to capsular PS type II and V, respectively (Singh and Srivastava, 2011; Moeller et al., 2021; Micoli et al., 2023). Furthermore, the recombinant protein termed TcdB_GT from *Clostridium difficile* was conjugated to its polysaccharide II (PSII) and induced similar anti-PSII IgG levels in mice, comparable to those induced by a CRM197-PSII conjugate. Simon et al. also proposed using the flagellin protein of *Salmonella* enteritidis as a carrier to conjugate with its OPS, thereby achieving enhanced protection through the additive effect of anti-O-antigen and anti-flagellin immune responses (Simon et al., 2011). Despite the fact that each new carrier protein needs to undergo testing for safety and efficacy, their development as scaffolds for next-generation glycoconjugates appears promising.

### 2.4 Metabolic engineering strategies to improve UDP-sugar supply

Uridine diphosphate (UDP)-sugars, such as UDP-glucose (UDPG), are crucial sugar precursors for the biosynthesis of important sugar-containing compounds, such as polysaccharides, glycoproteins, and glycolipids. These compounds are critical for cell growth and survival and are often limiting during recombinant biosynthesis (Feng et al., 2020). Therefore, it is crucial to ensure that their supply is sufficient *in vivo*. To address this issue, several regulatory schemes have been developed to improve the accumulation of endogenous UDP-sugars, such as the inhibition or knockout of non-essential pathways that consume UDP-sugars (Zhuang et al., 2017) and the fine-tuning of gene expression (Lv et al., 2019).

#### 2.4.1 Design and construction of an *Escherichia coli* glyco-platform to improve OPS production

As key precursors, UDP-sugars, especially UDPG, are involved in many cellular activities in *E. coli*, which can reduce their availability for OPS-based glycoconjugates biosynthesis (Verstrepen et al., 2004). Earlier studies have shown that supplementing large amounts of carbon sources, such as glucose, in the medium can alleviate the limitation of insufficient supply of UDPG (Liu S. et al., 2023). However, an excessive carbon source during the fermentation process can lead to overproduction of the acetic acid byproduct, which can ultimately lead to metabolic imbalance and inhibit the expression of recombinant enzymes (Pei et al., 2019). The main glucose-consuming pathways in *E. coli* are glycolysis and the PPP (Feng et al., 2020). Therefore, inhibiting multiple genes involved in these glucose-consuming pathways may have a positive effect on the production of OPS-based glycoconjugates (Simkhada et al., 2010; Pandey et al., 2013). Meanwhile, some studies have focused on using a mixed carbon source during the fermentation process, aiming to separate glycoside biosynthesis and cell growth (Soellner et al., 2013; Pei et al., 2019). This strategy was found to improve the overall titer, yield, and productivity of isoorientin generation (Wu et al., 2017; Tang et al., 2020).

To biosynthesize OPS-based glycoconjugates with high efficiency, the glycoengineering chassis was optimized by redirecting the carbon flux toward the biosynthesis of the required precursors (Wang et al., 2023a). To this end, *E. coli* K12 MG1655 was selected as the original strain, and multiple gene deletions were engineered in the genome to prevent carbon leakage from the pathway, thereby increasing the carbon flux toward OPS biosynthesis (Gleizer et al., 2019). Herein, Liu et al. established a synergistic glucose–glycerol co-feeding system to improve OPS accumulation by separating bacterial growth from polysaccharide biosynthesis. Specifically, *pfkA/B*, *zwf*, *nagB,* and *pykA/F* were blocked to inhibit or knockout non-essential pathways, such as the *E. coli* Embden-Meyerhof-Parnas pathway and the PPP that consume UDP-sugars (Jiang et al., 2015; Gleizer et al., 2019). Moreover, genes involved in the synthesis of ECA and the incomplete O16-specific OPS in *E. coli* MG1655 were also deleted to avoid interference with OPS production or to inhibit the consumption of the pool of essential substrates (Datsenko and Wanner, 2000; Yates et al., 2019). To enhance the glycerol consumption pathway and alleviate carbon catabolite repression, the gene *gldA*, encoding glycerol dehydrogenase, was also disrupted (Soellner et al., 2013). Overall, such a strategy can directly improve the reserve of UDP-sugar precursors and further increase OPS synthesis.

#### 2.4.2 Fine-tuning of gene expression to increase the supply of NDP-sugars

**Figure 4:**
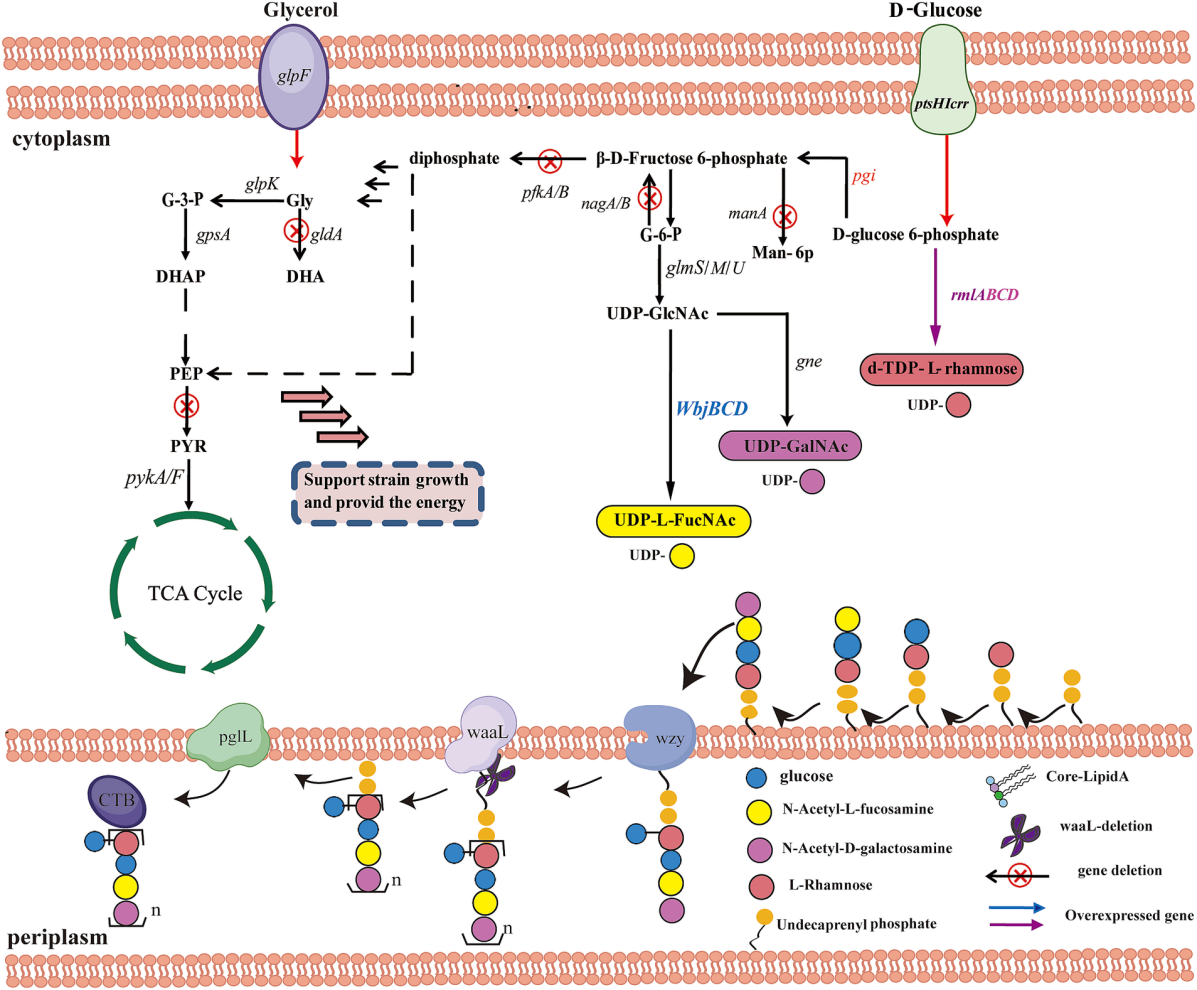
Schematic representation of a system in *E. coli* for dual-carbon utilization and orthogonal glycoprotein biosynthesis engineering. *ptsHICrr*, encoding phosphotransferase system (PTS); PEP, phosphoenolpyruvate; PYR, pyruvate; *pgi*, glucose-6-phosphate isomerase; *pgm*, phosphoglucomutase; *glmS*, glucosamine 6-phosphate synthase; *glmM*, phosphoglucosamine mutase; *glmU*, glucosamine 1-phosphate acetyltransferase/N-acetylglucosamine 1-phosphate uridyl transferase; UDP-GlcNAc, UDP-N-acetyl--D-glucosamine; *manA*, mannose-6-phosphate isomerase; Man-6P, D-mannose 6-phosphate; *pfkA*, 6-phosphofructokinase I; *pfkB*, 6-phosphofructokinase II; *nagB*, glucosamine 6-phosphate deaminase; *glpK*, glycerol kinase; *gldA*, glycerol dehydrogenase; *gpsA*, glycerol-3-phosphate dehydrogenase; G3P, glycerol 3-phosphate; DHAP, glycerone phosphate; *pykA/F*, pyruvate kinase II/I; *ppsA*, phosphoenolpyruvate synthetase; TCA cycle, Tricarboxylic acid cycle.

Efficient protein glycosylation of glycoconjugates in *E. coli* requires sufficient availability of polysaccharide precursors, prior to their transfer by OST to engineered carrier proteins (Ihssen et al., 2010). The most common strategy is to enhance the expression levels of native biosynthesis pathway genes for NDP-sugars or dNDP-sugars that can channel more glucose into these NDP-sugars or dNDP-sugars due to the elevated production of pathway enzymes (Hernández-Montalvo et al., 2003). The first step is to clone the gene cluster that expresses O-antigen by PCR into *E. coli* and further ensure the correct assembly of the glycan (Liu et al., 2017). Some studies have reported that the overexpression of the genes *pgm* and *galU1*, both of which are essential for UDPG biosynthesis, resulted in improved glucoside production (Weyler and Heinzle, 2015). Wang et al. (2023a) applied this strategy to significantly boost the levels of glycosyl donors (UDP-Glc, UDP-Gal, and UDP-GlcNAc) for monosaccharide building blocks present in the OPS of UPEC O21 cells (Figure 4). In their study, the genes *pgm*, *galU,* and *galE*, which are involved in the biosynthesis of UDP-Gal and UDP-Glc, were overexpressed. Furthermore, the genes *glmS*, *glmM,* and *glmU* were also overexpressed to boost the glycosyl donor UDP-GlcNAc (Deng et al., 2006). In such a system, this approach boosted the availability of UDP-sugars and glycosylation in the glycoengineering strain MGD15.

In another case, gene fine-tuning strategies were employed to promote OPS4 accumulation. Optimization of the pathway for enhancing dTDP-L-Rha and UDP-L-FucNAc synthesis can be targeted to improve glycosylation performance (Cress et al., 2014; Keinhörster et al., 2019). To identify enzymes with high catalytic activity, the biosynthetic pathways of dTDP-L-Rha and UDP-L-FucNAc from different bacterial sources were evaluated for efficient precursor production. Moreover, modular optimization was employed in this study by codon optimization (Alper et al., 2005). The biosynthetic pathways of dTDP-L-Rha and UDP-L-FucNAc from different bacterial sources were screened to identify enzymes with high catalytic activities to facilitate efficient precursor production (Ajikumar et al., 2010). Codon-optimized genes involved in the biosynthetic pathways of dTDP-L-Rha from *Mycobacterium tuberculosis* and *E. coli*, as well as those genes involved in the biosynthetic pathways of UDP-L-FucNAc from *P. aeruginosa*, have been studied (Sharon et al., 2012).

## 3 Current challenges in the field

To address the complexities, costs, and labor-intensive nature of traditional chemical and chemoenzymatic methods, the use of microbial cell factories has emerged as a promising alternative for the biosynthesis of OPS-based glycoconjugate vaccines (Weyant et al., 2018; Sorieul et al., 2023). However, there are several challenges that need to be addressed in the further application of microbial cell factories in synthesizing the desired glycoconjugate vaccines.

- The generation of multivalent glycoconjugates using cytoplasmic glycoconjugates presents unique challenges and complexities (Frasch, 2009). Creating multivalent glycoconjugates requires precise control over the number and arrangement of glycan chains attached to the protein (Bernardi et al., 2013). This requires not only specific glycosyltransferases for heterologous substrates but also strategies to control the density and pattern of glycosylation, which can significantly impact the immunogenicity and biological function of the resulting multivalent glycostructures (Clomburg et al., 2017).
- Polysaccharide heterogeneity produced by microbial cell factories presents challenges in the application of glycoconjugate vaccines (Huang and Wu, 2010). The size, branching, and composition of polysaccharides, whose biosynthesis in microbial cell factories can vary, contribute to this heterogeneity. Since the immunogenicity of polysaccharide antigens can vary based on their molecular weight, branching, and sugar composition，the heterogeneity in polysaccharide structures can significantly affect the quality and efficacy of glycoconjugate vaccines (Anish et al., 2021). Controlling the uniformity and length of polysaccharide structures is crucial for ensuring consistent vaccine performance and regulatory approval.
- The lack of structural information about glycosyltransferases limits their application and modification. Glycosyltransferases are multi-transmembrane proteins, which makes resolving their structures challenging. However, with the development of cryo-electron microscopy techniques, it is likely that more glycosyltransferase structures will be clearly resolved. This will greatly enhance our understanding of the functions of different structural domains within glycosyltransferases, and it holds promise for the artificial design and reconstruction of these domains. Such advancements could enable engineered enzymes to possess a more relaxed and extensive recognition capability for polysaccharide structures, as well as more precise glycosylation motifs, thereby laying the foundation for the development of multivalent conjugate vaccines using sets of orthogonal glycosyltransferases.
- The biosynthesis of polysaccharide-conjugate vaccines relies heavily on the bioinformatic analysis of bacterial polysaccharide antigen synthesis gene clusters and the establishment of molecular serotyping (Hu et al., 2013). However, deciphering polysaccharide antigens and conducting serotyping take time, thereby delaying the development of polysaccharide-based glycoconjugate vaccines and hindering the timely prevention and control of epidemic diseases.
- Most licensed glycoconjugate vaccines typically utilize traditional carrier proteins (Wilder-Smith, 2008; Micoli et al., 2018; Del Bino et al., 2022). Rational design and screening of novel carrier proteins are expected to further enhance the immunogenicity of glycoconjugate vaccines (Yue and Ma, 2015). The selection of new carrier proteins must adhere to some key principles for use in glycoconjugate vaccine development: a. The carrier protein should be produced in sufficient quantities, reliably and economically, with the appropriate degree of purity, to meet clinical requirements and allow for future commercial supply. b. It is essential for the carrier protein to be able to activate T-cells, thereby enhancing the overall immune response to the conjugate vaccine (Sun et al., 2019).
